# Associations between the Prenatal Diet and Neonatal Outcomes—A Secondary Analysis of the Cluster-Randomised GeliS Trial

**DOI:** 10.3390/nu11081889

**Published:** 2019-08-13

**Authors:** Julia Günther, Julia Hoffmann, Monika Spies, Dorothy Meyer, Julia Kunath, Lynne Stecher, Eva Rosenfeld, Luzia Kick, Kathrin Rauh, Hans Hauner

**Affiliations:** 1 Else Kröner-Fresenius-Centre for Nutritional Medicine, School of Medicine, Klinikum rechts der Isar, Technical University of Munich, Georg-Brauchle-Ring 62, 80992 Munich, Germany; 2 Competence Centre for Nutrition (KErn), Am Gereuth 4, 85354 Freising, Germany

**Keywords:** lifestyle intervention, pregnancy, dietary behaviour, neonatal outcomes, birth weight, large for gestational age (LGA), small for gestational age (SGA), obesity prevention

## Abstract

The prenatal lifestyle, including maternal dietary behaviour, is an important determinant of offspring health. This secondary cohort analysis of the GeliS (“healthy living in pregnancy”) trial investigated associations between antenatal dietary factors and neonatal weight parameters. The cluster-randomised GeliS trial included 2286 pregnant women. Dietary information was collected with food frequency questionnaires before or in the 12th (T0) and after the 29th week of gestation (T1). Consumption of vegetables (41.28 g per portion at T0, *p* = 0.001; 36.67 g per portion at T1, *p* = 0.001), fruit (15.25 g per portion at T1, *p* = 0.010) and dietary quality, measured with a Healthy Eating Index (39.26 g per 10 points at T0, *p* = 0.004; 42.76 g per 10 points at T1, *p* = 0.002) were positively associated with birth weight. In contrast, sugar-sweetened beverages (10.90 g per portion at T0, *p* = 0.003; 8.19 g per portion at T1, *p* = 0.047), higher sugar consumption at T0 (8.27 g per 10 g, *p* = 0.032) and early pregnancy alcohol intake (15.32 g per g, *p* = 0.039) were inversely associated with birth weight. Most other dietary factors were not associated with neonatal weight. Some components reflecting a healthy maternal diet were associated with a modest increase in offspring birth weight, whereas some unhealthy components slightly reduced neonatal weight.

## 1. Introduction

In recent decades, concern about childhood obesity has grown worldwide. The intrauterine environment has been found to have a profound impact on body weight and health development during childhood and adulthood. Offspring birth weight and weight-related parameters such as a high birth weight and large for gestational age (LGA) at birth have been suggested to modify the risk of future health consequences, including obesity [1] and metabolic diseases such as diabetes mellitus [2].

Maternal over-nutrition can result in excessive gestational weight gain (GWG) and has been found to increase the risk of a high birth weight by its influence on the intrauterine environment [3]. Ultimately, excessive GWG can increase the risk of childhood overweight and obesity [4,5] and may promote health complications in the offspring, including metabolic and cardiovascular diseases [6,7]. Several studies have investigated the specific role of the maternal diet in offspring growth and development [8,9]. While clear associations between the antenatal diet and offspring birth weight have been shown in malnourished women [10], the findings in well-nourished women were less conclusive. Although some components of the maternal diet have been suggested to influence the offspring’s birth weight including for instance, milk [11], fruit and vegetables [12] and fish [13], evidence in this field is sparse [8]. Comprehensive data on the impact of the overall antenatal maternal diet on neonatal growth parameters are lacking [14].

The importance of a healthy lifestyle during pregnancy, including adequate GWG, a healthy diet, and regular physical activity, has been increasingly recognised. Different approaches aiming to improve the antenatal maternal lifestyle have been developed. While some of these trials have been effective in reducing excessive GWG, data on diet and physical activity have been less often reported [15]. The large-scale cluster-randomised controlled GeliS study (“Gesund leben in der Schwangerschaft”/healthy living in pregnancy), which primarily aimed to reduce excessive GWG using a routine care approach, extensively reported on pregnancy and obstetric outcomes, as well as behavioural parameters after a lifestyle intervention [16]. Data on the effects of the intervention on GWG, as well as on maternal and infant outcomes, have recently been published [17]. The large GeliS cohort, recruited in the setting of routine prenatal care, collated a large and diverse maternal and offspring data set which provides a valuable opportunity to comprehensively analyse associations between maternal antenatal behaviour and infant growth and health parameters. This secondary analysis aims to investigate associations between the maternal diet during early and late pregnancy and neonatal outcomes in the GeliS cohort. The impact of mean daily intake of food groups, energy and macronutrients as well as dietary quality by means of a Healthy Eating Index (HEI) on offspring weight parameters at birth was elucidated herein.

## 2. Materials and Methods

### 2.1. Study Design

The cluster-randomised, controlled GeliS trial was conducted within the German routine care setting in medical practices. In five administrative regions of Bavaria, pairs of ten urban and rural areas were randomised into one intervention and one control area per pair. The randomisation process and study procedures have been described in detail in the published study protocol [16].

Study procedures were conducted in accordance with the declaration of Helsinki as well as with local regulatory requirements and laws. The study protocol was approved by the ethics committee of the Technical University of Munich and is registered at the ClinicalTrials.gov Protocol Registration System (NCT01958307).

### 2.2. Study Participants

Pregnant women were recruited before or in the 12th week of gestation in gynaecological and midwifery practices. Inclusion criteria were fulfilled if women were aged between 18 and 43 years, had a body mass index (BMI) between 18.5 and 40.0 kg/m^2^, had a singleton pregnancy, sufficient German language skills and provided written informed consent for participation in the trial. Women were excluded in case of a multiple or complicated pregnancy and if severe illnesses were diagnosed [16].

### 2.3. The Lifestyle Intervention Program

In the intervention group (IV), women attended a structured lifestyle intervention programme attached to routine prenatal care visits. They participated in three individual antenatal and one postpartum counselling sessions. Counselling included standardised information about an adequate GWG, a balanced diet and regular physical activity during pregnancy, and was conducted according to German recommendations [18]. Lifestyle counselling was performed by specifically trained midwives, gynaecologists and medical assistants in their practices.

Women in the control group (C) received routine prenatal care, complemented by a leaflet with general recommendations on a healthy antenatal lifestyle.

### 2.4. Study Outcomes

The primary outcome of the trial was the proportion of women showing excessive GWG as defined by the American Institute of Medicine (IOM) [19]. Data on the effect of the intervention on excessive GWG and some secondary outcomes have been published recently [17]. Despite a slight difference in offspring birth weight and length, no considerable between-group differences in maternal and neonatal outcomes were found [17]. Data from IV and C were thus pooled to form one cohort for the investigation of potential associations between the maternal diet and neonatal weight parameters.

### 2.5. Data Collection and Processing

Baseline characteristics of study participants were recorded by means of a screening questionnaire at study entry (≤12th week of gestation).

Maternal diet was assessed in both groups in early (≤12th week of gestation, T0) and late pregnancy (>29th week of gestation, T1) with food frequency questionnaires (FFQ). The self-administered FFQ was developed and validated by the Robert Koch Institute, Berlin, Germany for the “German Health Examination Survey for Adults” (DEGS) study [20] and was slightly modified for use in the GeliS trial. In the modified version, mean consumption frequency over the last four weeks was ranked by study participants from “never” to “more than five times per day” for 54 food items. Mean portion size was reported in standard measures such as cups, glasses, plates or bowls, and daily intake was calculated by combining frequency and portion size. If more than 20 out of the 54 food item questions were not answered, questionnaires were excluded from dietary analyses. Further, data of women reporting implausibly high daily intakes (either liquids >15 kg, or solid foods >10 kg, or both liquids >4 kg and solid foods >6 kg) were discarded due to over-reporting of food intake (according to personal communication by Dr. Gert Mensink, Robert Koch Institute, Berlin, Germany, 2018).

Recorded food items were summarised in food groups. Estimations of energy and macronutrient intake were made on the basis of information retrieved from the German food composition database (“Bundeslebensmittelschlüssel”, version 3.02, Max Rubner Institute (MRI), Karlsruhe, Germany) using OptiDiet PLUS software (version 6.0, GOE mbH, Linden, Germany). Some questions in the FFQ referred to food groups in sum and not to single food items. For these food groups, data of the German National Consumption Survey II (NVS II) assessing typical patterns in the consumption of food items within food groups were considered (personal communication from MRI, Federal Research Institute of Nutrition and Food, “Verzehrsmengen ausgewählter Lebensmittel aus der Nationalen Verzehrsstudie II” (NVS II), Karlsruhe, Germany, July 2018). Data of women were excluded from analyses of energy and macronutrient consumption if their estimated mean energy intake was below 4500 kJ or exceeded 20,000 kJ per day [21].

Dietary quality was rated by means of a Healthy Eating Index (HEI) specifically developed for the applied DEGS-FFQ from the Robert Koch Institute [22]. Intake of 14 food groups assessed with the FFQ was compared with the German Nutrition Society (DGE) recommendations for a healthy diet and scored from 0 (no adherence to recommendations) to 100 (high adherence to recommendations) points. Correspondingly, a HEI-score was calculated as mean from the group scores [22].

Information on birth weight and length was retrieved from birth records collected from medical practices. Neonatal weight below the 10th percentile for gestational age was defined as “small for gestational age” (SGA), weight above the 90th percentile for gestational age as “large for gestational age” (LGA). Offspring were considered to have a low birth weight if they weighed less than 2500 g and to have a high birth weight if they weighed more than 4000 g.

### 2.6. Statistical Analysis

Women were included in the analyses if dietary data on at least one assessment point as well as neonatal data (body weight and length) were available. Women dropping out of the study due to a miscarriage or pregnancy termination, severe pregnancy complications or maternal death were excluded from the analyses. IV and C were pooled to form one cohort. Baseline characteristics are presented as mean ± standard deviation or proportion if appropriate.

For the analysis of associations between dietary variables (as mean daily portion or percent of energy if appropriate) and continuous offspring outcomes (birth weight, BMI), linear regression models were applied. Portions sizes were defined according to the FFQ for the different food items. For the analysis of dichotomous outcomes (SGA, LGA, low birth weight, high birth weight), binary logistic regression models were applied. Regression models were adjusted for group assignment, maternal pre-pregnancy age, BMI category and parity. Results are presented as effect sizes or odds ratios with 95% confidence intervals. *p*-values below 0.05 were considered as statistically significant. All analyses were conducted using SPSS software (IBM SPSS Statistics for Windows, version 24.0, IBM Corp, Armonk, NY, USA).

## 3. Results

### 3.1. Study Participants and Baseline Characteristics

**Figure 1:**
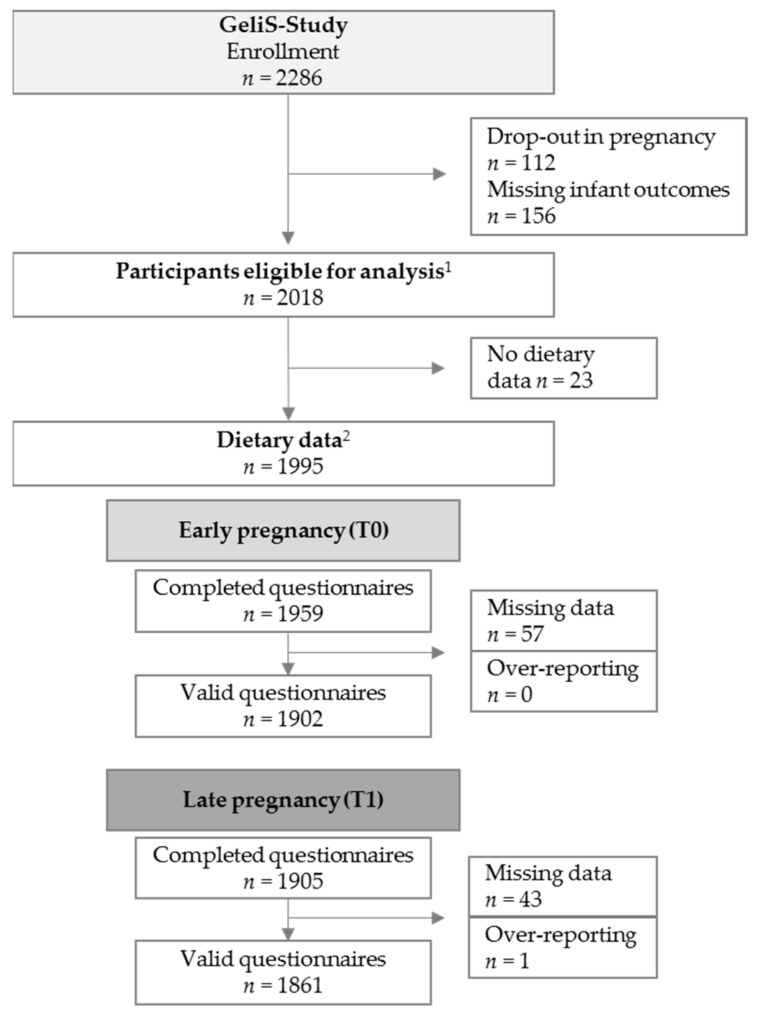
Participant flow. ^1^ Women without miscarriages, late loss of pregnancy, terminations, pregnancy complications that interfered with the intervention, maternal deaths and/or lack of infant outcomes (birth weight, birth length). ^2^ Women who provided dietary data at T0 or T1. T0: Dietary assessment before or in the 12th week of gestation; T1: Dietary assessment after the 29th week of gestation.

Altogether, 2286 women were recruited for participation in the GeliS trial. A total of 112 women dropped out during the course of pregnancy (Figure 1). Women were further excluded from cohort analyses if they did not provide the relevant infant parameters (*n* = 156), resulting in a total of 2018 eligible participants. The maternal and neonatal characteristics of this sample are shown in Table 1. Dietary intake data were provided by 1995 of the 2018 women. After exclusion of invalid questionnaires and over-reporters, dietary data of 1902 (T0) and 1861 (T1) women were analysed in relation to neonatal weight parameters.

### 3.2. Associations between the Maternal Diet and Infant Weight and Weight-Related Parameters

Associations between antenatal food intake and neonatal birth weight and BMI are shown in Table 2. Associations with BMI z-scores are shown in Supplementary Table S1. There was evidence for a positive association between maternal fruit and vegetable consumption and infant birth weight (Table 2). An increase in offspring weight at birth by 41.28 g (95% CI 17.87 to 64.70 g, *p* = 0.001) was observed per portion of vegetables (150 g) in early pregnancy, and by 36.67 g/portion in late pregnancy (95% CI 15.95 to 57.39 g, *p* = 0.001). These observations were also reflected in the BMI of the neonates (T0: *p* = 0.017, T1: *p* = 0.002). A significant association between fruit intake and birth weight and BMI was found for the late pregnancy dietary assessment (birth weight: 15.25 g increase per portion of fruit (95% CI 3.67 to 26.83 g, *p* = 0.010), BMI: 0.04 kg/m^2^ increase per portion (95% CI 0.01 to 0.07, *p* = 0.009)), but not for early pregnancy (birth weight: *p* = 0.322, BMI: *p* = 0.172). In contrast, maternal consumption of sugar-containing beverages was significantly inversely associated with weight at birth. In early pregnancy, one glass of soft drinks (200 mL) consumed by the mother reduced offspring birth weight by 10.90 g (95% CI −18.17 to −3.64 g, *p* = 0.003). In late pregnancy, there was evidence of a reduction by 8.19 g (95% CI −16.26 to −0.11 g, *p* = 0.047) per consumed glass of soft drinks. The association between soft drink intake and weight was not reflected in the BMI of the neonates (T0: *p* = 0.076, T1: *p* = 0.437). For the other food groups, such as dairy products, fast food and meat, no significant association between the consumed amount and offspring weight and BMI could be shown, either in early or in late pregnancy (Table 2).

Table 3 shows associations between antenatal food intake and infant weight-related outcomes. Fruit consumption in early pregnancy was significantly associated with increased odds of having a LGA neonate (OR 1.08 per portion, 95% CI 1.02 to 1.15, *p* = 0.016). Moreover, fast food consumption in early pregnancy but not in late pregnancy increased the odds of the offspring weighing > 4000 g at birth (T0: OR 3.14 per 250 g portion, 95% CI 1.26 to 7.84, *p* = 0.014).

There was no evidence for an association between energy or macronutrient intake as percent of energy and offspring birth weight and BMI (Table 4). With increasing saccharose intake (per 10 g of sugar) in early pregnancy, a slight reduction in birth weight by 8.27 g (95% CI −15.83 to −0.70 g, *p* = 0.032) was observed. There was no statistical evidence for such an association in late pregnancy (*p* = 0.074). Similarly, alcohol consumption in early but not late pregnancy was related to a reduction in offspring weight at birth by 15.32 g per g (95% CI −29.83 to −0.80 g, *p* = 0.039).

### 3.3. Diet Quality and Infant Weight Outcomes

Maternal dietary quality, measured with a HEI, was positively associated with birth weight in both early (39.26 g per 10 points (95% CI 12.29 to 66.22 g, *p* = 0.004)) and late pregnancy (42.76 g per 10 points (95% CI 15.98 to 69.55 g, *p* = 0.002)). Additionally, late pregnancy HEI was positively associated with the neonatal BMI (*p* = 0.006), whereas there was no evidence for such an association with early HEI (*p* = 0.081). These observations did not translate into increased odds of the offspring being LGA or having a birth weight above 4000 g (Table 5). Per 10 g maternal sugar consumption in early pregnancy, the odds of the offspring being born with a low birth weight were increased by 1.07 (95% CI 1.01 to 1.13, *p* = 0.026). Maternal energy intake, as well as macronutrient intake as percent of energy, did not show any further associations with the offspring’s risk of being born with low or high birth weight, SGA or LGA (Table 5).

## 4. Discussion

This secondary analysis of the GeliS trial shows associations between the maternal diet in early and late pregnancy and offspring weight parameters. Although no consistent associations between energy intake and macronutrient composition and neonatal birth weight and weight-derived parameters were shown, several aspects of maternal dietary behaviour were related to neonatal weight parameters.

Our analysis provides evidence that a healthy balanced antenatal diet, indicated by higher scores on the HEI, promotes birth weight within an adequate range and does not increase the risk of high birth weight or LGA. An increase in neonatal body weight with an increasing HEI has also been reported by others [25]. We could not confirm findings by investigators who observed that women with a healthier dietary pattern bore offspring with a reduced risk for low birth weight [26]. However, the latter study was performed in Ghana where the risk of low birth weight is much higher compared to developed countries [27]. Moreover, fruit and vegetable consumption were identified as factors that are positively associated with infant weight and BMI at birth. This observation is in line with findings of other studies reporting an increase in birth weight with increasing intake of fruits and vegetables [12,28]. While maternal vegetable intake did not modify the risk of being born SGA or LGA in our study, a high fruit intake in early pregnancy seemed to slightly increase the risk of LGA.

Our data did not indicate a beneficial association between the consumption of fruits and vegetables and the odds of being born SGA. In general, the data showing a protective effect of vegetable and fruit consumption on infants being born SGA is conflicting [12]. This may result from the heterogeneity in methods used for dietary assessment in the few studies. Moreover, effects may differ between high- and low-income countries [12]. The observed effects of fruit and vegetable intake in our cohort may be a surrogate reflecting healthier dietary choices, as similar associations were observed regarding the HEI.

For instance, factors related to a lower neonatal body weight in the GeliS cohort included food parameters reflecting a rather unhealthy diet, such as soft drinks, sugar and alcohol intake. Soft drink and saccharose consumption were inversely associated with birth weight. An increase in sugar consumption in early pregnancy was additionally found to increase the risk of being born with a low birth weight. Both soft drinks and saccharose are energy-providing components of the diet that do not deliver any further nutrients of specific value. A high consumption may be accompanied by a lower consumption of nutritious foods and this could explain the negative association with birth weight. A negative effect of the intake of sugar and sugar-rich products on offspring birth weight has also been reported in other studies [28,29], whereas the role of soft drinks needs to be further elucidated. In the literature, the consumption of sugar-containing beverages has been suggested to have both an increasing [14] and a decreasing effect [30] on neonatal birth weight. Another factor, which we found to be negatively associated with birth weight, was alcohol consumption. This is consistent with evidence from a recent review and meta-analysis suggesting a relationship between low amounts of alcohol intake and the prevalence of children born SGA [31]. Although alcohol intake was found to be associated with a reduction in birth weight in the GeliS trial, there was no evidence for a modification of the SGA risk. However, reported alcohol consumption was generally low among our study population [32] and intake may be underreported, thus limiting the validity of our observations as well as those from others.

Fast food consumption was associated with an increased risk of the offspring being born with a high birth weight. This may be related to the high energy density of fast food which can generally increase overall energy intake [33]. A higher risk for delivering macrosomic infants in women eating a diet rich in energy-dense fast food has been suggested [34], but evidence is sparse. In our cohort, we found no evidence for an effect of energy and fat intake itself on birth weight and weight-related parameters. Likewise, although other maternal dietary factors, such as the intake of dairy products [11] or fish [13], have previously been shown to modify infant birth weight, we were not able to confirm these observations. 

The presented cohort data have some limitations. Due to the high number of recruited pregnant women, maternal diet was assessed by means of a self-administered FFQ and energy and macronutrient intake were estimated based on the FFQ. More precise methods for the assessment of the maternal diet, such as weighted dietary records, were not feasible in the context of the large-scale GeliS study. However, they may have a greater potential to reveal significant effects of the maternal diet on neonatal weight and weight-related characteristics. In addition, maternal antenatal physical activity was not considered as a covariate in exploring associations between food intake and offspring weight data. We acknowledge that physical activity may be an additional modifying factor for food and energy intake and may likewise influence neonatal outcomes [35]. Due to the nature of the trial designed as a routine care study, offspring data were limited to reported body weight and weight-derived parameters. An additional assessment of infant body composition may provide a more comprehensive picture of the observed associations, as suggested for instance in the INFAT trial [36]. Moreover, infant weight was measured at different clinics, and it is possible that measurements may not have been conducted completely standardised. 

The reported effects of the maternal diet on infant weight and weight-related parameters at birth were only modest and clinical relevance of the observed associations is thus questionable. Nonetheless, this secondary analysis of the GeliS cohort has some strengths that merit particular attention. A wide spectrum of dietary data was analysed with regard to offspring weight and weight-related data, including the consumption of particular foods and food groups, energy and macronutrient intake, as well as dietary quality measured with an HEI that was specifically developed for the FFQ used in this study [20,22]. Within the large-scale GeliS trial, we collected dietary and infant data directly in the setting of routine prenatal care. We were able to follow participants from early pregnancy through the postpartum period. In contrast to many other studies, dietary data were collected twice during pregnancy in the GeliS cohort, enabling separate analyses of potential effects of the antenatal diet on offspring outcomes in early and late pregnancy. Our cohort included both primi- and multiparous women from different BMI categories with varying educational levels. This offered a view on the impact of the maternal diet on offspring weight in a diverse target population which is, in many characteristics, representative for German women of childbearing age. 

## 5. Conclusions

In conclusion, a healthy maternal diet characterised by a high intake of fruit and vegetables, as well as a high HEI, was shown to modestly increase birth weight within the adequate range without augmenting the risk for high birth weight or LGA. In contrast, intake of soft-drinks, saccharose and alcohol represented factors associated with a decrease in birth weight. Maternal intake of fast food in early pregnancy was related to a higher risk for infants to have a high birth weight. In the on-going follow-up of the GeliS cohort, the long-term effects of the maternal diet on offspring weight development during infancy will be evaluated.

## Figures and Tables

**Table 1:** Characteristics of study participants eligible for cohort analyses.

Maternal and Neonatal Characteristics	Total (*n* = 2018)
Maternal characteristics	
Pre-pregnancy age, years	30.3 ± 4.4 ^a^
Pre-pregnancy weight, kg	68.2 ± 13.4 ^a^
Pre-pregnancy BMI, kg/m^2^	24.4 ± 4.5 ^a^
Pre-pregnancy BMI category	
BMI 18.5–24.9 kg/m^2^	1311/2018 (65.0%)
BMI 25.0–29.9 kg/m^2^	464/2018 (23.0%)
BMI 30.0–40.0 kg/m^2^	243/2018 (12.0%)
Educational level	
General secondary school	320/2014 (15.9%)
Intermediate secondary school	856/2014 (42.5%)
(Technical) High school	838/2014 (41.6%)
Country of birth	
Germany	1790/2014 (88.9%)
Others	224/2014 (11.1%)
Further maternal characteristics	
Nulliparous	1162/2018 (57.6%)
Living with a partner	1939/2011 (96.4%)
Full-time employed	1056/1996 (52.9%)
Gestational diabetes mellitus ^b^	209/1940 (10.8%)
Pregnancy hypertension ^c^	161/2015 (8.0%)
Preterm delivery ^d^	131/2016 (6.5%)
Neonatal characteristics	
Birth weight, g	3337.6 ± 517.8 ^a^
Birth length, cm	51.3 ± 2.6 ^a^
Head circumference, cm	34.7 ± 1.6 ^a^
BMI, kg/m^2^	12.7 ± 1.3 ^a^
SGA	172/2016 (8.5%)
LGA	148/2016 (7.3%)
Low birth weight	101/2018 (5.0%)
High birth weight	169/2018 (8.4%)

^a^ mean ± SD. ^b^ diagnosed with a 2-h oral glucose tolerance test at weeks 24–28 of gestation according to national and international standards [23,24]. ^c^ Systolic blood pressure > 140 mmHg or diastolic blood pressure > 90 mmHg at at least two time points. ^d^ < 37 weeks of gestation. BMI: body mass index; SGA: Small for gestational age; LGA: Large for gestational age.

**Table 2:** Associations between maternal food intake and offspring birth weight and body mass index.

Food Groups	Birth Weight (g)		BMI (kg/m^2^)	
	Adjusted Effect Size ^a^ (95% CI)	Adjusted *p* Value ^a^	Adjusted Effect Size ^a^ (95% CI)	Adjusted *p* Value ^a^
Soft drinks ^b^ (200 mL/day)				
T0	−10.90 (−18.17, −3.64)	0.003	−0.02 (−0.03,0.00)	0.076
T1	−8.19 (−16.26, −0.11)	0.047	−0.01 (−0.03,0.01)	0.437
Light drinks ^c^ (200 mL/day)				
T0	−5.70 (−17.59,6.19)	0.347	−0.00 (−0.03,0.03)	0.919
T1	−5.89 (−18.34,6.55)	0.353	0.01 (−0.02,0.04)	0.577
Vegetables (150 g/day)				
T0	41.28 (17.87,64.70	0.001	0.07 (0.01,0.13)	0.017
T1	36.67 (15.95,57.39)	0.001	0.09 (0.03,0.14)	0.002
Fruit (150 g/day)				
T0	5.55 (−5.44,16.55)	0.322	0.02 (−0.01,0.05)	0.172
T1	15.25 (3.67,26.83)	0.010	0.04 (0.01,0.07)	0.009
Dairy products (200 g/day)				
T0	8.41 (−5.60,22.41)	0.239	0.00 (−0.03,0.04)	0.838
T1	3.13 (−9.37,15.62)	0.624	0.02 (−0.01,0.05)	0.233
Meat and meat products (150 g/day)				
T0	6.79 (−54.36,67.94)	0.828	0.00 (−0.15,0.15)	0.993
T1	3.35 (−60.86,67.55)	0.919	0.00 (−0.16,0.17)	0.966
Sweets and snacks (50 g/day)				
T0	11.69 (−8.07,31.44)	0.246	0.00 (−0.05,0.05)	0.895
T1	−4.97 (−21.12,11.18)	0.547	0.00 (−0.04,0.05)	0.855
Fast food (250 g/day)				
T0	9.08 (−153.36,171.52)	0.913	0.08 (−0.32,0.48)	0.702
T1	−95.15 (−273.51,83.22)	0.296	−0.17 (−0.63,0.29)	0.470

Estimated is the regression coefficient describing the association between the intake of a portion of a food item or food group and infant weight and BMI. Portion sizes are defined according to the applied food frequency questionnaire. ^a^ linear regression models adjusted for pre-pregnancy BMI, age, parity and group assignment. ^b^ sugar-containing sweetened beverages. ^c^ low or non-caloric sweetened beverages. BMI: body mass index; T0: assessment before or in the 12th week of gestation; T1: assessment after the 29th week of gestation.

**Table 3:** Associations between maternal food intake and neonatal weight-related parameters.

Food Groups	Low Birth Weight		High Birth Weight		SGA		LGA	
	Adjusted Odds Ratio ^a^ (95% CI)	Adjusted *p* Value ^a^	Adjusted Odds Ratio ^a^ (95% CI)	Adjusted *p* Value ^a^	Adjusted Odds Ratio ^a^ (95% CI)	Adjusted *p* Value ^a^	Adjusted Odds Ratio ^a^ (95% CI)	Adjusted *p* Value ^a^
Soft drinks ^b^ (200 mL/day)								
T0	1.04(0.99,1.09)	0.150	0.95(0.88,1.02)	0.149	1.03(0.99,1.08)	0.123	0.94(0.87,1.02)	0.155
T1	1.01(0.94,1.09)	0.766	0.95(0.88,1.03)	0.221	1.00(0.94,1.07)	0.973	0.95(0.87,1.03)	0.212
Light drinks ^c^ (200 mL/day)								
T0	0.98(0.87,1.11)	0.778	0.99(0.91,1.08)	0.858	1.01(0.93,1.10)	0.783	1.00(0.92,1.09)	0.971
T1	1.01(0.90,1.14)	0.864	1.01(0.94,1.10)	0.763	1.05(0.98,1.12)	0.213	1.02(0.94,1.10)	0.627
Vegetables (150 g/day)								
T0	0.85(0.67,1.08)	0.184	1.09(0.94,1.27)	0.262	0.98(0.84,1.16)	0.841	1.13(0.96,1.32)	0.138
T1	0.81(0.63,1.05)	0.113	1.14(1.00,1.31)	0.058	0.88(0.74,1.05)	0.146	1.14(0.98,1.32)	0.081
Fruit (150 g/day)								
T0	1.05(0.96,1.14)	0.284	1.06(0.99,1.13)	0.094	0.97(0.89,1.05)	0.460	1.08(1.02,1.15)	0.016
T1	1.07(0.96,1.18)	0.224	1.05(0.97,1.14)	0.197	0.90(0.81,1.01)	0.063	1.06(0.97,1.14)	0.187
Dairy products (200 g/day)								
T0	0.95(0.82,1.10)	0.458	1.04(0.95,1.14)	0.387	1.05(0.96,1.14)	0.294	1.06(0.97,1.16)	0.177
T1	0.98(0.85,1.13)	0.772	0.95(0.85,1.06)	0.321	0.95(0.84,1.06)	0.343	0.99(0.90,1.10)	0.903
Meat and meat products (150 g/day)	
T0	1.40(0.87,2.25)	0.172	1.20(0.82,1.76)	0.349	0.85(0.54,1.33)	0.474	1.16(0.77,1.72)	0.481
T1	1.52(0.82,2.82)	0.183	1.25(0.79,1.98)	0.347	1.00(0.61,1.63)	0.998	1.19(0.73,1.94)	0.486
Sweets and snacks (50 g/day)								
T0	1.07(0.91,1.25)	0.423	1.02(0.90,1.17)	0.722	0.97(0.84,1.12)	0.683	1.06(0.93,1.20)	0.424
T1	1.14(0.99,1.30)	0.066	0.96(0.84,1.09)	0.496	1.00(0.88,1.13)	0.963	0.93(0.81,1.07)	0.308
Fast food (250 g/day)								
T0	1.35(0.36,5.09)	0.655	3.14(1.26,7.84)	0.014	1.10(0.37,3.25)	0.862	2.31(0.85,6.33)	0.103
T1	1.87(0.34,10.36)	0.473	2.21(0.64,7.61)	0.210	1.68(0.47,5.97)	0.427	1.04(0.26,4.16)	0.955

Estimated is the regression coefficient describing the association between the intake of a portion of a food item or food group and infant weight-related parameters. Portion sizes are defined according to the applied food frequency questionnaire. ^a^ linear regression models adjusted for pre-pregnancy BMI, age, parity and group assignment; ^b^ sugar-containing sweetened beverages; ^c^ low or non-caloric sweetened beverages. BMI: body mass index; LGA: large for gestational age; SGA: small for gestational age; T0: assessment before or in the 12th week of gestation; T1: assessment after the 29th week of gestation.

**Table 4:** Associations between maternal energy, macronutrient intake and dietary quality and offspring birth weight and body mass index.

Energy and Macronutrient Intake	Birth Weight (g)		BMI (kg/m^2^)	
	Adjusted Effect Size ^a^ (95% CI)	Adjusted *p* Value ^a^	Adjusted Effect Size ^a^ (95% CI)	Adjusted *p* Value ^a^
Energy [100 kcal/day]				
T0	−0.04 (−3.89,3.80)	0.984	−0.01 (−0.02,0.00)	0.224
T1	3.15 (−0.45,6.76)	0.087	0.01 (−0.00,0.02)	0.218
Carbohydrates [10 E%]				
T0	−8.69 (−39.35,21.97)	0.579	0.01 (−0.06,0.09)	0.769
T1	9.16 (−22.26,40.58)	0.568	0.01 (−0.07,0.09)	0.764
Saccharose [10 g/day]				
T0	−8.27 (−15.83,−0.70)	0.032	−0.02 (−0.04,0.00)	0.074
T1	3.72 (−3.90,11.34)	0.339	0.01 (−0.01,0.03)	0.271
Protein [10 E%]				
T0	48.88 (−28.19,125.95)	0.214	0.08 (−0.12,0.27)	0.439
T1	14.36 (−60.91,89.64)	0.708	0.08 (−0.11,0.27)	0.425
Fat [10 E%]				
T0	5.35 (−33.39,44.08)	0.787	−0.03 (−0.13,0.06)	0.489
T1	−17.00 (−55.49,21.50)	0.387	−0.04 (−0.14,0.06)	0.458
Alcohol [g]				
T0	−15.32 (−29.83,−0.80)	0.039	−0.01 (−0.05,0.02)	0.439
T1	−40.67 (−105.97,24.63)	0.222	−0.11 (−0.28,0.05)	0.182
Caffeine [100 mg]				
T0	−6.57 (−32.54,19.41)	0.620	−0.03 (−0.10,0.03)	0.313
T1	−12.20 (−40.04,15.64)	0.390	−0.05 (−0.13,0.02)	0.139
HEI [10 points]				
T0	39.26 (12.29,66.22)	0.004	0.06 (−0.01,0.13)	0.081
T1	42.76 (15.98,69.55)	0.002	0.10 (0.03,0.16)	0.006

Estimated is the regression coefficient describing the association between the intake of a certain amount or percentage and infant weight and BMI. ^a^ linear regression models adjusted for pre-pregnancy BMI, age, parity and group assignment. BMI: body mass index; HEI: Healthy Eating Index; T0: assessment before or in the 12th week of gestation; T1: assessment after the 29th week of gestation.

**Table 5:** Associations between maternal energy, macronutrient intake and dietary quality and neonatal weight-related parameters.

Energy and Macronutrient Intake	Low Birth Weight		High Birth Weight		SGA		LGA	
	Adjusted Odds Ratio ^a^ (95% CI)	Adjusted *p* Value ^a^	Adjusted Odds Ratio ^a^ (95% CI)	Adjusted *p* Value ^a^	Adjusted Odds Ratio ^a^ (95% CI)	Adjusted *p* Value ^a^	Adjusted Odds Ratio ^a^ (95% CI)	Adjusted *p* Value ^a^
Energy [100 kcal/day]								
T0	1.01 (0.98,1.05)	0.560	1.01 (0.98,1.04)	0.509	1.00 (0.97,1.03)	0.854	1.01 (0.98,1.04)	0.630
T1	0.99 (0.95,1.03)	0.594	1.01 (0.98,1.03)	0.659	0.98 (0.96,1.01)	0.267	0.99 (0.96,1.02)	0.604
Carbohydrates [10 E%]								
T0	0.96 (0.73,1.26)	0.761	0.94 (0.76,1.17)	0.587	0.92 (0.74,1.13)	0.408	1.03 (0.83,1.29)	0.780
T1	1.05 (0.76,1.45)	0.786	1.01 (0.79,1.27)	0.966	0.83 (0.65,1.06)	0.131	1.09 (0.85,1.39)	0.501
Saccharose [10 g/day]								
T0	1.07 (1.01,1.13)	0.026	0.97 (0.92,1.03)	0.358	1.01 (0.96,1.07)	0.627	0.99 (0.94,1.05)	0.702
T1	0.98 (0.90,1.07)	0.664	0.98 (0.92,1.04)	0.513	0.96 (0.90,1.02)	0.210	0.96 (0.90,1.03)	0.244
Protein [10 E%]								
T0	0.77 (0.38,1.55)	0.457	1.31 (0.76,2.23)	0.331	0.95 (0.56,1.61)	0.852	1.02 (0.58,1.81)	0.940
T1	1.11 (0.51,2.40)	0.802	1.32 (0.76,2.30)	0.329	0.98 (0.55,1.75)	0.948	1.49 (0.84,2.66)	0.173
Fat [10 E%]								
T0	1.14 (0.80,1.61)	0.467	1.05 (0.80,1.38)	0.735	1.14 (0.88,1.49)	0.329	0.94 (0.71,1.25)	0.673
T1	0.91 (0.61,1.36)	0.647	0.92 (0.69,1.23)	0.584	1.33 (0.99,1.79)	0.057	0.79 (0.58,1.07)	0.131
Alcohol [g]								
T0	1.01 (0.88,1.15)	0.905	0.77 (0.55,1.07)	0.115	1.05 (0.98,1.13)	0.181	1.03 (0.94,1.12)	0.555
T1	1.05 (0.53,2.07)	0.887	0.85 (0.46,1.58)	0.616	0.74 (0.35,1.58)	0.442	0.81 (0.42,1.59)	0.544
Caffeine [100 mg]								
T0	1.09 (0.88,1.34)	0.431	0.94 (0.77,1.14)	0.494	1.03 (0.86,1.24)	0.739	1.03 (0.87,1.22)	0.718
T1	0.93 (0.68,1.27)	0.638	0.84 (0.66,1.07)	0.154	1.13 (0.93,1.37)	0.210	0.87 (0.68,1.10)	0.245
HEI [10 points]								
T0	0.86 (0.68,1.09)	0.220	1.11(0.92,1.34)	0.290	0.84(0.70,1.02)	0.076	1.10(0.90,1.35)	0.363
T1	0.80 (0.61,1.06)	0.117	1.15(0.94,1.41)	0.171	0.85(0.69,1.04)	0.104	1.15(0.93,1.43)	0.188

Estimated is the regression coefficient describing the association between the intake of a certain amount or percentage and infant weight-related parameters. ^a^ linear regression models adjusted for pre-pregnancy BMI, age, parity and group assignment. BMI: body mass index; HEI: Healthy Eating Index; LGA: large for gestational age; SGA: small for gestational age; T0: assessment before or in the 12th week of gestation; T1: assessment after the 29th week of gestation.

## Supplementary Materials

The following are available online at https://www.mdpi.com/2072-6643/11/8/1889/s1, Table S1: Associations between dietary parameters and offspring body mass index z-score at birth.
