# Supplementary material for: Associations between the Prenatal Diet and Neonatal Outcomes—A Secondary Analysis of the Cluster-Randomised GeliS Trial
Source: Nutrients. 2019 Aug 13;11(8):1889. doi: 10.3390/nu11081889 (PMC6723267; doi:10.3390/nu11081889)
Supplement: Supplementary file 1 [file nutrients-11-01889-s001.pdf]

Table S1. Associations between dietary intake parameters and offspring body mass index z-score at birth.

Food groups	BMI z-score[a] Adjusted effect size[b] (95% CI)	BMI z-score[a] Adjusted p value[b]
**Soft drinks[c] (200 ml/day)**		
T0	-0.01(-0.03,0.00)	0.069
T1	-0.01(-0.02,0.01)	0.401
**Light drinks[d] (200 ml/day)**		
T0	-0.00(-0.03,0.03)	0.912
T1	0.01(-0.02,0.04)	0.621
**Vegetables (150 g/day)**		
T0	0.06(0.01,0.10)	0.016
T1	0.07(0.03,0.11)	0.001
**Fruit (150 g/day)**		
T0	0.01(-0.01,0.04)	0.199
T1	0.03(0.01,0.06)	0.009
**Dairy products (200 g/day)**		
T0	0.00(-0.02,0.03)	0.775
T1	0.02(-0.01,0.04)	0.217
**Meat and meat products (150 g/day)**		
T0	0.00(-0.12,0.12)	0.962
T1	0.01(-0.13,0.14)	0.923
**Sweets and snacks (50 g/day)**		
T0	0.00(-0.04,0.04)	0.925
T1	0.00(-0.03,0.04)	0.862
**Fast food (250 g/day)**		
T0	0.07(-0.25,0.39)	0.672
T1	-0.13(-0.49,0.24)	0.499
**Energy [100 kcal/day]**		
T0	-0.01(-0.01,0.00)	0.219
T1	0.01(-0.00,0.01)	0.201
**Carbohydrates [10 E%]**		
T0	0.01(-0.06,0.07)	0.867
T1	0.01(-0.06,0.07)	0.811
**Saccharose [10 g/day]**		
T0	-0.01(-0.03,0.00)	0.064
T1	0.01(-0.01,0.03)	0.265
**Protein [10 E%]**		
T0	0.07(-0.08,0.22)	0.359
T1	0.07(-0.08,0.23)	0.364
**Fat [10 E%]**		
T0	-0.02(-0.10,0.05)	0.544
T1	-0.03(-0.11,0.05)	0.469
**Alcohol [g]**		
T0	-0.01(-0.04,0.02)	0.466
T1	-0.09(-0.23,0.06)	0.247
**Caffeine [100 mg]**		
T0	-0.03(-0.08,0.03)	0.309
T1	-0.05(-0.11,0.01)	0.104
**HEI [10 points]**		
T0	0.05(-0.00,0.10)	0.071
T1	0.08(0.02,0.13)	0.006

Estimated is the regression coefficient describing the association between the intake of a portion of a food item, food group or dietary parameter and infant BMI z-score. Portion sizes are defined according to the applied food frequency questionnaire. [a]BMI z-scores were calculated based on German standards [1]. [b]linear regression models adjusted for pre-pregnancy BMI, age, parity and group assignment. [c]sugar-containing sweetened beverages. [d]low or non caloric sweetened beverages. BMI: body mass index; T0: assessment before the 12th week of gestation; T1: assessment after the 29th week of gestation.

1. Kromeyer-Hauschild, K.; Wabitsch, M.; Kunze, D.; Geller, F.; Geiß, H.C.; Hesse, V.; von Hippel, A.; Jaeger, U.; Johnsen, D.; Korte, W.; et al. Perzentile für den Body-mass-Index für das Kindes-und Jugendalter unter Heranziehung verschiedener deutscher Stichproben. *Mon. Kinderheilkd* **2001**, *149*, 807–818, doi:10.1007/s001120170107.
